# Austrian Pharmacy in the 18^th^ and 19th Century

**DOI:** 10.3797/scipharm.1004-06

**Published:** 2010-06-15

**Authors:** Christa Kletter

**Affiliations:** Department of Pharmacognosy, University of Vienna, Althanstraße 14, 1090, Vienna, Austria

**Keywords:** History of Pharmacy, Austria, Apothecary, Pharmaceutical education

## Abstract

This overview reflects the extensive changes in the health care system which had significant effects on the apothecary’s profession and education. In the 18^th^ century Maria Theresia assigned Gerard van Swieten to modernize the medical curriculum and to work out reforms for health care. The resulting sanitary bill released in 1770 and amended in 1773 became effective for the whole empire and influenced greatly the apothecary’s profession. The Viennese Medical Faculty continued to be the supervisory body for the apothecaries, a situation which prolonged the conflicts between the faculty and the apothecaries. The financial and social distress prevalent in the 19^th^ century also affected the apothecary business and led to a crisis of the profession. Furthermore, the apothecaries’ missing influence over the sanitary authorities delayed the release of a badly needed new apothecary bill until 1906. The introduction of a specific pharmaceutical curriculum at the university in 1853 was a great step forward to improve the pharmaceutical education. Nevertheless, the secondary school exam was not compulsory for the studies until 1920 and, therefore, the graduates were not on a par with other university graduates before that date. Women, except nuns, were not allowed to work as pharmacists until 1900.

## Introduction

If you refer to Austria of the 18^th^ century, you may raise the question “What was Austria considered to be at that time? Austria was a monarchy under Habsburg rule with Vienna as its cultural and political centre. At the beginning of the 18^th^ century the empire encompassed the territories of today’s Austria (excluding Salzburg), Hungary, as well as Bohemia, Moravia, Silesia, regions which belong today to the Czech Republic and Poland, also territories of contemporary Slovenia, Croatia, Romania, Northern Italy and Southern Germany. In the course of the 18^th^ century various regions were annexed to the empire: the Grand Duchy of Tuscany; the Kingdom of Naples; the Banat; Galicia and Bukovina, territories which belong today to Poland and the Ukraine; Northern Serbia; the Small Wallachia; and the Austrian Netherlands forming today the territories of Luxembourg and Belgium. Thus, the empire included far-away regions with diverse cultural and ethnic backgrounds. Some territories were again lost in the course of the 18^th^ century: the Austrian Netherlands, Silesia, regions of Northern Italy, the Kingdom of Naples, regions of Southern Germany, Northern Serbia and the Small Wallachia [1].

At the beginning of the 18^th^ century there were no uniform regulations regarding the apothecary’s profession and no unitary health system in the empire. Apothecary ordinances were decreed for particular towns or regions only. Health care measures, for example against epidemics, were taken in case of onset, precautions did not exist. In general, the pharmacies were located in town, because it was the only place a solvent clientele existed. The poor people in the countryside took advantage of medicines distributed free of charge by religious orders or relied on traditional herbal medicine or bought obscure remedies from quack doctors that roamed the streets.

## Apothecary Ordinances

Since the 16^th^ century ordinances regulating the education and profession of the apothecary existed in Vienna, due to its position as the centre of the empire. Later ordinances were effective not only for Vienna but also for the whole Duchy of Austria above and below the river Enns, regions which relate to the provinces of contemporary Vienna, Lower and Upper Austria. [2]

At the beginning of the 18th century the ordinance released in 1666 for Vienna, Lower and Upper Austria was still in force. At that time the apothecaries had the freedom of the city. To become an apothecary the young man first had to do a five-year-long apprenticeship in a pharmacy [2]. After having completed this training, he generally left this pharmacy and travelled around seeking work in different pharmacies to enlarge his knowledge. If he ultimately wanted to acquire a pharmacy in the Duchy of Austria he needed to pass an exam before the Medical Faculty of the University of Vienna, which also administered his apothecary oath as the respective supervisory body.

Apothecary ordinances existed not only in Vienna but also in other cities of the empire. Several such ordinances were based on the Viennese ordinance or on the ordinance released for the Duchy of Austria. One foreign ordinance, for example, the ordinance of Augsburg, had served as a directive for the concept of an ordinance for Innsbruck in the early 17^th^ century. On the other hand, some towns had no apothecary orders at all, such as Graz in Styria [3]. And a special set of regulations were applied, for instance, to the apothecaries of Prague. [4]

## The Importance of the Viennese Medical Faculty

Since its founding – the statutes of the Medical Faculty of the University of Vienna were issued in 1389 – the Medical Faculty regarded itself as the superior health care institution in the Duchy. Several privileges decreed by bishops and sovereigns for the Medical Faculty gave the latter an increasing influence on the apothecaries, which was limited to Viennese pharmacies in the early days. As the Viennese apothecaries refused to comply with the supervision and the criminal law exercised by the Faculty, disputes dragged on for decades between the Medical Faculty, the Viennese town council and the Viennese apothecaries. The apothecaries’ opposition to the Medical Faculty’s supervision was ultimately in vain, because the faculty exerted more influence on the authorities and could push through its interests. The faculty not only wanted to assert its position of power over the apothecaries but also tried to eliminate problems in the pharmacies and to implement regulations for the consistent preparation of high quality medicines [5].

## Maria Theresia’s Concern for Health Care

At the turn of the 18^th^ century Austria’s health care system was underdeveloped in comparison to the countries of Western Europe. Maria Theresia, the ruler of Austria between 1740 and 1780, recognised this deficit and wanted to improve the situation. Her interest in improvement originated, on one hand, from her feeling of responsibility for the people of her empire but, on the other hand, also from her concern to maintain the labour force of the population. Her ambitions were to improve the health care system and to set up a new study plan for medicine.

Looking for an appropriate personality for such an undertaking, she addressed the Catholic Gerard von Swieten, a native-born Dutch and disciple of Herman Boerhaave, a famous medical teacher at the renowned University of Leiden. It was not surprising that Maria Theresia sent for a famous doctor from the United Netherlands as her personal physician and reformer for her health care system, because, in the 18^th^ century, the United Netherlands were very progressive in comparison to the Central European territories of the Habsburgs. Despite his initial reluctance to Maria Theresia’s proposal, van Swieten finally accepted her invitation and arrived at the Viennese Court in 1745 [6, 7].

## Van Swieten’s Impact on Health Care in Austria

According to his initial contract, van Swieten held the position of a personal physician to Maria Theresia and the Imperial Family, he was Protomedicus (the Chief Physician of the Court) and the prefect of the Court Library. However, he quickly gained the full confidence of the sovereign. The different unsalaried functions that he had taken over since 1749 show clearly the great influence that he had already gained in health care. He was promoted to superior supervisor for civil and military health care issues of the empire, to Dean of the Medical Faculty of the University of Vienna, and to president of the court commissions responsible for censorship and studies. He fervently advocated the improvement of medical education at the University of Vienna. By appointing foreign doctors with great medical knowledge as professors at the University, he was able to force open the rigid medieval structures of the University and to replace the backward medical training in Vienna with the more modern medicine taught in Western Europe. He fulfilled his task of reforming medical education and establishing a centralist public health care system to the utter satisfaction of Maria Theresia. The law on which his reforms were based was the so-called “Sanitätshauptnormativ” or “Generalsanitätsnormativ”, a sanitary bill put into force in 1770. In 1773 a supplement to this bill was released which contained very important passages regarding the apothecary issues. This extensive sanitary bill implemented the first uniform health care system in the empire and applied to all territories of the Habsburg Empire [8, 9].

## Van Swieten – the Apothecary

Gerard van Swieten was not only educated as a medical doctor, he also had full training as an apothecary. He started his career as an apothecary in 1715 when he became an apprentice in a pharmacy in Amsterdam. Some years later he worked as a fully trained apothecary in Leiden, where he was admitted as a member of the Collegium Pharmaceuticum. In 1717, he started his university career when he first enrolled in the philosophy faculty of the University of Leiden. Only later he changed to the medical faculty, where he finished his medical studies in five years. This was far above the average time of two years of study. As van Swieten was an excellent student, the most feasible explanation for his many years of study might have been his concurrent profession as an apothecary. He needed his apothecary work as a means of subsistence but he also recognised it as a sensible supplement to his medical studies [10].

## Van Swieten’s Influence on the Apothecary’s Education

In the course of his reforms at the University of Vienna van Swieten provoked some outstanding changes in the medical curriculum, which was also for the benefit of the apothecary’s education. He installed a new chair for botany and chemistry and appointed Robert Laugier as first professor for this professorship in 1749. Laugier’s lectures in botany and chemistry were not only held for students of medicine but were also compulsory for pharmacy apprentices and journeymen according to van Swieten’s instructions. In earlier apothecary ordinances only the length of the apprenticeship, personal qualifications such as knowledge of reading, writing and Latin, creed or legitimate birth were considered to be important. Those ordinances did not include any directives for academic education. The compulsory university lectures for the pharmacy personnel decreed by van Swieten as well as some regulations included in the “Sanitätshauptnormativ” of 1770 and its supplement of 1773 focussed on improving the theoretical knowledge of the prospective apothecaries. In this sanitary bill of 1770 one important paragraph focussed on education, saying that each owner of a pharmacy had to be examined at a university located within the territory of the Habsburg Empire. To take the exam at the University of Vienna was an advantage for the examinee, because this allowed him to acquire a pharmacy at any place in the whole empire [4].

One of the outstanding professors at the University of Vienna was Nikolaus Joseph von Jacquin, who succeeded Robert Laugier in the chair of botany and chemistry. Jacquin was a renowned scientist whose main scientific interest was in the field of botany. Among others, he published major volumes devoted to the flora of Austria, the West Indies and the Americas, which contained artistically valuable illustrations of plants. His lectures in chemistry were particularly important for the apothecary’s education, because he met their specific needs in this field. Jacquin was deeply involved in developing the new pharmacopoeia, the so-called “Pharmacopoea Austriaco-Provincialis”, which was released in 1774. It replaced the Viennese Dispensatory, which had been in force since 1729. For the first time, this new pharmacopoeia applied to all territories of the Habsburg Empire. In 1794, an improved edition of the Pharmacopoea Austriaco-Provincialis was released and used until 1812. The Pharmacopoea Austriaco-Provincialis had a modern pharmacopoeia concept. It listed the plants and animals according to Linné’s nomenclature and the improved edition followed Lavoisier’s system of chemical nomenclature. In addition, obsolete recipes had been eliminated and the number of recipe constituents reduced [11].

Van Swieten’s influence on the apothecary system was based not only on the founding of a chair of botany and chemistry but also on his function as Dean of the Medical Faculty of the University of Vienna. He was entrusted with the superintendence of the inspections carried out each year in the Viennese pharmacies. He also was the head of the examination board for the apothecaries’ exams. Since 1637/38 all journeymen in Vienna and in the Duchy above and below the Enns River were requested to take the apothecary examination before the Medical Faculty in Vienna. Before having set up the chair of botany and chemistry in 1749, all theoretical knowledge had to be acquired in private studies. Since that time an improvement in the apothecary education was brought about by van Swieten’s call for compulsory attendance of the University lectures by the pharmacy apprentices and journeymen [4].

## The Viennese Apothecary Board

In 1782 Vienna had 11 apothecaries who were organised in a board. There are some indications that a community of interest had already existed in earlier times. Expressions such as “senior” of the apothecaries are known from the Middle Ages. An explicit sign that an apothecary board had already existed in the first half of the 18^th^ century are remaining board meeting minutes dating back to 1723. The foundation of apothecary boards in the entire empire was finally decreed in 1773 in the amendment to the sanitary bill of 1770.

The Viennese apothecary board was disbanded in 1782 by Emperor Joseph II, Maria Theresia’s son and successor as sovereign. The disbandment of the board resulted from a scandal in connexion with a delivery of falsified and inferior remedies for the Austrian army. Joseph II was, on one hand very angry about this incident, but, on the other hand, he took this opportunity to disband the board and clear the way for setting up badly needed new pharmacies in Vienna. Consequently, parallel to the board disbandment, Joseph II disclosed that “each properly trained and examined apothecary may be allowed to open a pharmacy in or outside the city walls of Vienna” [12]. In the same year the emperor put the Viennese Medical Faculty in charge of the pre-inspections of such newly founded pharmacies [13].

This liberalization of pharmacy foundations by assigning a personal right to an appropriate person to open a pharmacy led to an increase in pharmacies, particularly outside the city walls of Vienna, and to greater competitive pressure among the Viennese apothecaries. Due to the lack of an apothecary board, the Medical Faculty of Vienna could again assure its right of supervision. In 1794, the Medical Faculty was authorized to assess the professional quality of each candidate who wanted to open a pharmacy by a personal concession [14].

As the missing apothecary board had negative effects on the Viennese pharmacies, Emperor Franz II reintroduced the apothecary board in 1796 and decreed concurrently a new ordinance for the apothecary board. This ordinance not only regulated the rights and duties of the apothecaries but also included new instructions for the apothecary examinations. According to this ordinance, the influence of the Viennese Medical Faculty as supervisor of the apothecaries had declined, at least regarding the exams. Hence, the two presidents of the board and two selected apothecaries gave the exams. The Medical Faculty was not represented anymore by physicians but only by the notary of the Medical Faculty, who acted as chairman of the commission. In this ordinance the education of the apprentice was also more clearly specified. Complying with this bill, they had to attend the university lectures and take exams about them. The apothecary board was entrusted with more rights. The board now had the right to evaluate the knowledge of the apprentice and to decide over his promotion to journeyman [15]. The ordinance for the Viennese apothecary board not only influenced the Viennese pharmacies but also affected other apothecary board ordinances in different parts of the empire.

## The Question of the Creed

The possibility to start one’s career as an apothecary was greatly influenced by the creed of the candidate. As early as 1644, it was compulsory for the apothecary and his apprentice to follow the catholic religion [2]. In 1678 this restriction was eased for those apprentices following the protestant religion who could show credibly that they would convert in due time to Catholicism [16]. In contrast, the employment of a journeyman did not necessarily depend on his catholic beliefs [2]. Later, in the sanitary bills of 1770 and 1773, no more religious restrictions were imposed [17].

Despite the restrictions for non-Catholics, it must have been possible for Jews to acquire a pharmacy at that time. They had a bad reputation which was even recorded in an apothecary privilege decreed in 1671 by Emperor Leopold. This privilege contained the information that a lot of evil comes from Jewish pharmacies, because they sell harmful medicines. Therefore, they are not allowed to dispense medicines to Christian people. Only among themselves were they allowed to distribute their medicines. Maria Theresia amended and annotated this privilege in 1748, but the passage regarding the Jewish pharmacies was neither changed nor commented [18]. Maria Theresia was a devout catholic and had a negative attitude toward Jews, which she revealed in some of her legal orders. Van Swieten seemed to have a similar approach because he, for example, disapproved of Jews at the university [19]. Later, under the rule of Joseph II the policy regarding Jews changed and even affected the apothecary business. For example, in 1783 a Jew of Prague received the privilege by Joseph II to run a pharmacy [20]. However, in 1829 an explicit interdiction for Jews to run pharmacies was put into force [21]. This restriction had lasted for 31 years and was only overruled in 1860 by an order of the Ministry of the Interior [22]. The Jews finally got all rights of Austrian citizens by the constitutional law of 1867, which granted all citizens equal rights, irrespectively of their creed [23].

## Introduction of a Pharmaceutical Curriculum at the University

In the 19^th^ century the apothecaries had to meet new challenges such as the rapid development in chemistry and the introduction of new synthetic drugs. The apothecary’s task no longer consisted predominantly of the manufacture of remedy ingredients but in the use of new analytical methods to identify and secure the quality of the substances which were bought from chemical companies. However, the apothecaries of the Habsburg Empire were poorly prepared to meet those new requirements. The lectures in chemistry were still held by physicians of the Medical Faculty who could not communicate the necessary special knowledge. Any alterations in the curriculum for pharmacists were not considered of primary importance and, therefore, the education of the apothecaries was only modified when changes in medical studies occurred.

In 1804 new academic regulations for medicine did away with all private studies, a situation which also had consequences for the apothecaries. From then on, all journeymen had to attend a one-year course at the university if they wanted to be admitted to take the examination for apothecary. This specific course treated the topics special natural history, botany and chemistry. In subsequent decades lectures changed and new ones were added, but the lectures still did not comply with the growing importance of chemistry. In 1849, the lectures in natural science were finally transferred from the Medical Faculty to the Philosophy Faculty [4].

Another step towards independence of pharmacy from the medical curriculum came in 1853, when a special curriculum for pharmacy was issued. The criteria established for admission to the study were as follows: attendance of the first four classes of secondary school, completion of the apothecary’s apprenticeship, and two years of work as journeyman or assistant as he was called at that time. The length of the study was two years ending with the degree of a master of pharmacy (Magister der Pharmazie). Since that time pharmacy could be studied in Vienna, Graz, Innsbruck, Prague, Pest, Krakow and Lemberg. Despite this new curriculum, the students of pharmacy were not admitted as regular students, because they had not taken the final secondary school examination. This was also unfavourable for any career in civil service, because, due to this missing examination, the master of pharmacy was not regarded as a full academic degree [4]. Only since 1920 has this final secondary school examination been the pre-condition for the study of pharmacy in Austria [24].

In the middle of the 19^th^ century, the study of pharmacy was only compulsory if a person wanted to buy a pharmacy shop or if he applied for the position of a “Provisor”, a kind of manager of a pharmacy under specific conditions. The degree was expensive and, therefore, many assistants could not afford it. On the other hand, not all assistants who had completed the degree were able to purchase a pharmacy, because the costs were high and the number of pharmacies limited. This situation resulted in two types of pharmacy assistants: academically qualified ones and those without academic training.

## The Profession Crisis in the 19^th^ Century

In the early 19^th^ century the population suffered from great financial distress as a consequence of the wars between Austria and France. Furthermore, the shortage in foreign medicinal plants and the increasing practice of medical doctors of prescribing medication with few and inexpensive ingredients as well as the business competition of herbalists led to financial distress also among the apothecaries. The assistants had to suffer from social hardships such as excessive working hours, low payment, crowded housing conditions, and a lack of health care, accident insurance and retirement plans.

In the first half of the 19^th^ century censorship regulations were very strict, opinions or critical comments on whatever topic could not be freely expressed and the founding of associations was looked at with mistrust. Therefore, the deprived assistants had little chance to discuss their problems in public or even in professional associations. After the revolution in 1848 and the liberalization of daily life, numerous new pharmaceutical associations were registered in order to provide a platform for discussion and to give financial support to severely deprived colleagues.

Similarly, the pharmacy owners were very unsatisfied with their professional situation. In 1848, the general discontent was articulated at an all-Austrian conference convened by the Viennese apothecary board. The following issues, which mainly concerned the owners’ interests were considered as particularly urgent [25]:

- no increase in pharmacies beyond the actual demand
- assignment and inheritance of apothecary privileges and licenses without restrictions
- no more underbidding when supplying medicine for public institutions
- publishing of a new tariff list
- professional representation of the apothecaries before all medical authorities
- improvement in the pharmaceutical education
- publishing of a new pharmacopoeia
- release from the oppressive subordination exerted by the medical authorities

The demands of the apothecaries died away unheard, because the apothecary board had little influence on the health care authorities. The only success was the release of a new pharmacopoeia.

In 1861 “the General Austrian Apothecary’s Association”, Allgemeiner Österreichischer Apotheker-Verein”, was registered to back up the owners and assistants, because the board had failed to achieve their aims. In the first years after its founding, the association addressed mainly the questions of the concession system, the tariff list and the problem of the insufficient pharmaceutical education. To better the apprentices’ education the association founded a school to convey the necessary knowledge. In 1865 the courses started. They lasted one year, later one and a half years. The attendance was free of charge and voluntary. As of 1905 the courses were compulsory for Viennese apprentices or Aspirants, as they were now called. The school closed only in 1922 after a new curriculum for the university study had been released [26].

Throughout the entire 19^th^ century the endeavours of the apothecaries to get new professional regulations which could meet their demands were without success. As of 1860 the trade law was not effective for the pharmacies, new apothecary regulations were of primary importance [27]. At least, from 1861, two paragraphs of the trade law which referred to the transfer of the business after the owner’s death became also effective for the pharmacies [28]. In 1870 a new sanitary bill came into force, but the needs of the apothecaries were disregarded [29]. In 1890, a ministerial order which was an addendum to the study reform of 1889 provoked a new crisis. This order stated that exclusively persons who had finished the study of pharmacy were allowed to be employed as assistants in the pharmacies [30]. Discussions started among the apothecaries, because they feared a shortage of pharmaceutical personnel and higher wages due to this regulation. There were also uncertainties if assistants without diploma could be still employed in pharmacies. Two years later an order of the same ministry clarified this question by admitting the employment of assistants without academic degree. However, those assistants lost all rights to take over a pharmacy [31].

At the same time, in 1891 a group of assistants founded the “General Austrian Apothecary Assistant Association”, “Allgemeiner Österreichischer Apotheker-Assistentenverein”. The founding session was kept secret because the initiators feared they would lose their jobs if their identities were revealed. The aims were clearly oppositional to the interests of the pharmacy owners [32].

- support of deprived colleagues
- legitimate representation of the assistants in all pharmaceutical bodies
- reform of the apothecary board ordinance
- reform of the concession system to facilitate the acquisition of pharmacies
- job placement free of charge

Even the social aspect was stressed, because the founders of the association also demanded arranging of convivial evenings.

All these activities initiated new heated debates and the health care authorities were finally forced to raise this issue and conduct conversations with all concerned bodies. After tedious discussions over some years a bill was passed in 1906, which represented a new pharmacy law. A fundamental issue of this law was the call for founding an apothecary chamber with separate professional representations for pharmacy owners and assistants. However, the concession system remained unchanged [33]. The problems of social security regarding pensions after retirement, for widows and orphans were solved in the same year due to a new bill released by the emperor [34]. Nevertheless, the upcoming First World War and the political events following the collapse of the Habsburg Empire delayed the founding of a new apothecary chamber until 1947.

## Women in Pharmacy

Today, the pharmaceutical profession is a woman’s domain. But where were the women throughout the centuries? Until the end of the 19^th^ century, the apothecary profession was male dominated. Women appeared at the best as apothecary wives. They were not allowed to start an apprenticeship in a pharmacy. Nevertheless, nunneries were granted the privilege to employ females as apothecaries in their pharmacies. Such nun apothecaries could occasionally even serve their apprenticeship in a public pharmacy [35]. In the second half of the 19^th^ century things started to change and there was increasing public discussion about higher education of women. While in the Austrian part of the dual monarchy Austria-Hungary the spectre of a female apothecary was still discussed fervently, in Hungary, in 1895, women were already being admitted for the study of pharmacy at the University of Budapest [36]. According to reports from the Jagiellonian University in Krakow, two ladies, Hedwig Sikorska-Klemensiewicz and Ianina Kosmowska, were admitted to pharmaceutical studies and the final examinations because of special permissions granted by the competent authorities in Vienna. They graduated as pharmacists as early as 1898 [37].

Finally, in 1900, an order of the Ministry of the Interior allowed all women in Austria to be admitted to the pharmaceutical profession and to enrol in the study of pharmacy [38]. Neither the outcry of the male pharmacists nor unobjective comments in pharmaceutical journals hindered the women in conquering this male profession irrevocably. The first reported Austrian female pharmacist who graduated after the release of this order was Friederike Reich. She received her diploma at the University in Lemberg, a town which belongs today to Ukraine [39].

To close this overview about the development of pharmacy in the Austrian Empire during the 18^th^ and 19^th^ century it can be stated that the release of the sanitary bill of 1770 and its amendment of 1773, the introduction of a pharmaceutical curriculum in 1853, the study reforms of 1889 and 1922 and the apothecary law enacted in 1906 contributed greatly to the acceptance of pharmacists as recognized professionals with a solid academic background.
